# Expression of the Receptor for Advanced Glycation End Products, a Target for High Mobility Group Box 1 Protein, and its Role in Chronic Recalcitrant Rhinosinusitis with Nasal Polyps

**DOI:** 10.1007/s00005-014-0325-7

**Published:** 2014-12-12

**Authors:** Karolina Dzaman, Miroslaw J. Szczepanski, Marta Molinska-Glura, Antoni Krzeski, Mariola Zagor

**Affiliations:** 1 Division of Dentistry, Department of Otolaryngology, Medical University of Warsaw, Stepinska 19/25, 00-739 Warsaw, Poland; 2 Department of Computer Science and Statistics, University of Medical Sciences, Poznan, Poland; 3 Department of Clinical Immunology, University of Medical Sciences, Poznan, Poland

**Keywords:** Nasal polyposis, Innate immunity, Chronic inflammation, RAGE, HMGB1

## Abstract

A receptor for advanced glycation end products (RAGE) and its ligand high mobility group box 1 (HMGB1) protein has been linked to several chronic diseases, and acts as a trigger for inflammation signaling. Here, we study RAGE and HMGB1 expression in chronic, recalcitrant rhinosinusitis with nasal polyps (CRSwNP) to determine its potential clinical significance, i.e., disease recurrence and severity. RAGE and HMGB1 expression in CRSwNP was evaluated by immunohistochemistry in epithelial cells of fresh sinonasal mucosa samples obtained from the patients diagnosed with recalcitrant CRSwNP (*n* = 25) and normal control mucosa (NC) (*n* = 26). RAGE and HMGB1 expression levels in tissues were correlated with disease severity assessed by nasal endoscopy, CT scan, number of previous sinus surgeries, allergy status and nasosinusal microbiology. RAGE and HMGB1 were moderately or strongly expressed in CRSwNP tissue. No or weak RAGE expression was found in NC. HMGB1 was equally strongly expressed in NC. We observed a strong correlation between RAGE and disease severity, recurrence, undergone operations, asthma and aspirin exacerbated respiratory disease (AERD). Elevated RAGE expression is associated with increased disease severity, as well as allergy and AERD in patients with recalcitrant CRSwNP. It is possible that the explanation for recurrent CRSwNP pathogenesis might be related to RAGE overexpression with subsequent sinus mucosa hyperproliferation, necessitating several operations.

## Introduction

Recent studies suggest that the receptor for advanced glycation end products (RAGE) has been implicated in initiating and perpetuating inflammatory responses. Due to an enhanced level of RAGE ligands in chronic disorders, this receptor is hypothesized to have a causative effect in a range of inflammatory diseases (Bassi et al. 2008; Singh et al. 2014). RAGE is a multiligand receptor and a member of the immunoglobulin superfamily of cell surface molecules which is found on smooth muscle cells, macrophages, endothelial cells and astrocytes. Its name comes from its ability to bind advanced glycation end products (AGE) (Singh et al. 2014). Most characterized RAGE ligands are either released during cell stress (S100 proteins, high mobility group box 1 (HMGB1) protein and nucleic acids), or generated during prolonged hyperglycemia and inflammation (AGE, amyloid) (Sims et al. 2010). Some authors characterize RAGE as a cell surface receptor that influences the concentration threshold at which DNA activates inflammatory responses in vitro and in vivo (Sirois et al. 2013). RAGE binds directly to DNA and RNA, and promotes their uptake into cells, lowering the immune recognition threshold for activation of Toll-like receptor 9 (TLR9), the principal DNA-recognizing transmembrane signaling receptor. A higher level of nucleic acid concentration, such as during infections or in situations of increased cell damage, can trigger signaling via RAGEs and their downstream inflammatory effects. RAGE can thereby sensitize cells to extracellular nucleic acids.

Recent studies suggest that the interaction between RAGE and its ligand HMGB1 has been linked to several chronic diseases (Yang et al. 2013). HMGB1-RAGE interaction provides an extracellular trigger for inflammatory cell proliferation, migration and survival (Erlandsson Harris and Andersson 2004; Raucci et al. 2007; Sims et al. 2010; Ulloa and Messmer 2006; Yang et al. 2013). HMBG1 also has the capacity to bind other molecules, such as lipopolysaccharides and to induce maturation of dendritic cells via activation of TLR4 (Youn et al. 2008). The pathogenesis of persistent inflammation is hypothesized to include ligand binding, upon which RAGE signals activate the nuclear factor (NF)-κB. NF-κB controls several genes involved in inflammation. RAGE itself is upregulated by NF-κB. These interactions trigger the activation of key signaling pathways involved in the regulation of innate and adaptive immunity (Andersson and Tracey 2004b).

Notwithstanding this evidence, and the fact that RAGE and HMGB1 have been proposed as a novel therapeutic target for infectious and inflammatory disorders (Andersson and Erlandsson-Harris 2004; Andersson and Tracey 2004a, 2011; Mantell et al. 2006; Schierbeck et al. 2011), no data have been published concerning the expression and function of RAGE and HMGB1 in recalcitrant nasal polyposis.

Nasal polyps (NPs) are a very common inflammatory disease classified as a subtype of chronic sinusitis, chronic rhinosinusitis with nasal polyps (CRSwNP) (Fokkens et al. 2012). The prevalence of NPs is increased in patients diagnosed with cystic fibrosis, asthma, non-steroidal anti-inflammatory drug intolerance aspirin exacerbated respiratory disease (AERD), Churg-Strauss syndrome, or sarcoidosis. Although the source of inflammation may be variable, researchers theorize that these inciting events lead to disruption of the epithelial lining and initiate a resultant inflammatory cascade (Fokkens et al. 2012). If this inflammation does not subside in its normal timely fashion, stromal edema consolidates and may result in polyp formation (Norlander et al. 1996).

Because the pathway that leads to the formation of NPs has not been completely elucidated, effective long-term treatments remain difficult to pinpoint. Those patients who showed recurrence of NPs despite surgery and a postoperative regimen including topical steroids are classified as recalcitrant CRSwNPs. Patients without endoscopic evidence of polyps at least 6 months after surgery are classified as responsive CRSwNPs (Reh et al. 2010). Novel therapeutic targets are, therefore, needed for developing new medical therapies for recalcitrant CRSwNP. We hypothesize that RAGE and its ligand, HMGB1, could play a critical role in NPs. RAGE-mediated augmentation of inflammation can be deregulated in immune pathologies. RAGE, therefore, represents an attractive target for pharmacological intervention. The present study investigates the expression of RAGE and HMGB1 in epithelial cells of sinonasal mucosa (SM) obtained from the patients with recalcitrant CRSwNP vs. normal controls (NC) and discusses the role of RAGE and HMGB1 in the pathogenesis of recalcitrant rhinosinusitis and disease severity.

## Materials and Methods

### Tissues

Fresh sinus mucosa samples from the patients diagnosed with CRSwNP were obtained during surgery from the ostiomeatal complex and stored. Patients were subsequently classified as either treatment responsive or treatment recalcitrant, based on the long-term outcomes of medical and surgical therapy. Patients without endoscopic evidence of NPs at least 6 months after surgery were classified as responsive CRSwNPs and excluded from the study. Those patients who showed recurrence of NPs despite surgery and a postoperative regimen including topical steroids were classified as recalcitrant CRSwNPs (*n* = 25) and included in the study. The CRSwNPs patients included 13 males and 12 females (median age: 47 years; range 24–77 years). The mucosa samples were obtained from ostiomeatal complex region: the uncinate process or lateral surface of middle nasal concha if the patient had had surgery before. As NC, patients with nasal structural deformities without clinical and radiological evidence of chronic rhinosinusitis were included (*n* = 26). NC samples included non-inflammatory changed normal mucosa from the uncinate process of the ostiomeatal complex region. The group of NC comprised 18 men and 8 women (median age: 40 years; range 16–69 years). Table 1 summarizes characteristics of the patients included in this study. The study was in accordance with the ethical standards of the Local Ethics Committees of Warsaw Medical University in Poland (No. KB 103/2012) and with the Helsinki Declaration. All participants signed an informed consent.

**Table 1:** Clinicopathological characteristics of NC patients and CRSwNP patients included in this study

Characteristics	NC patients (*n* = 26)	CRSwNP patients (*n* = 25)	Statistical analysis
Sex			
Male	18	13	
Female	8	12	
Age			
Range	16–69	24–77	
Median	40	47	
Allergy	2	10	*p* = 0.009
Asthma	0	8	*p* = 0.004
AERD	2	11	*p* = 0.020
Average CT Lund-Mackay scores	0.8	14.6	
CT scores			
0–4	26	0	
5–11	0	8	
12–18	0	8	
19–24	0	9	
Average value IgE IU/mL	60	129	
Positive microbiology	25 %	45 %	

The patients included in the study received no oral steroids 2 months before surgery

*AERD* aspirin exacerbated respiratory disease

### Disease Severity Measure

The classification of disease severity was performed using endoscopic appearance scores—Lund polyp staging system (Lund and Kennedy 1995) and CT scan graded by Lund-Mackay scoring system (Fokkens et al. 2012; Lund and Kennedy 1997). We also recorded the number of sinus surgeries that had been performed before our surgery as a reflection of the recalcitrance of the NPs.

### Disease Etiology

#### Allergy Status

In looking for etiology of NPs, allergy status was assessed based on a medical interview, a skin prick test and a total IgE level in patients’ blood samples. AERD and asthma were diagnosed based on spirometry and medical anamnesis, according to the global initiative for asthma (GINA) criteria.

#### Microbiology

To determine the nasosinusal microbiology, regardless of the presence of mucopus, middle meatus cultures were obtained under endoscopic control followed by agar culture.

#### Cytology

To determine the population of inflammatory cells in the nasal cavity, cytology was taken endoscopically under the middle turbinate. The cells were smeared on glass slides, fixed with alcohol, stained with hematoxylin-eosin (H+E) and evaluated under a light microscope (Tarchalska-Krynska et al. 1993).

### Immunohistochemistry

The surgically removed tissues were fixed in 10 % formaldehyde and sections stained with H+E were prepared and evaluated by light microscopy. The following primary antibodies were used for immunostaining of tissue sections: rabbit polyclonal anti-human HMGB1 (Abcam, Cambridge, UK), rabbit polyclonal anti-human RAGE (LifeSpan Bioscience Inc., Seattle, WA, USA) and isotype control IgG (Dako, Gdynia, Poland). Paraffin sections of SM tissues and control-SM were stained as previously described (Szczepanski et al. 2009). After standard deparaffinization, the EnVision+System (Dako) was used for staining according to the manufacturer’s instructions. In short, after an overnight incubation with the antibodies, sections were first incubated with labeled polymer-horseradish peroxidase anti-rabbit antibody and then with 3,3′-diaminobenzidine. To eliminate non-specific binding of the secondary antibody, tissue sections were incubated with a serum-free protein blocker before adding the primary antibodies. Sections were counterstained with Meyer’s hematoxylin and mounted in resin. Slides were evaluated in a light microscope (magnification: 200× or 400×). For digital image analysis, the software AnalySIS^B was used. All stained sections were analyzed and scored by two independent investigators (M. J. S. and K. D.) to avoid bias, and the two scores were averaged and recorded. The sections were scored according to the percentage of rhinosinusitis tissue staining positively for HMGB1 or RAGE (<25 % = 0; 25–75 % = 1; and >75 % = 2). The level of staining intensity was recorded as 0: none, 1: weak, 2: moderate, or 3: strong. For the better understanding of RAGE and HMGB1 expression, intensity and positivity staining were combined and shown as a RAGE/HMGB1 ratio. As positive control tissues for HMGB1 and RAGE staining, the intestine and kidney were used, respectively.

### Statistical Analysis

Data were summarized by descriptive statistics. Fisher’s exact tests were used to determine if there was a difference in HMGB1 and RAGE expression among tissue types. Adjustments to *p* values were made using the Bonferroni step-down procedure. The ANOVA Kruskala-Wallisa test was used to evaluate differences between CRSwNP patients and NC.

## Results

### Cytology

**Fig. 1:**
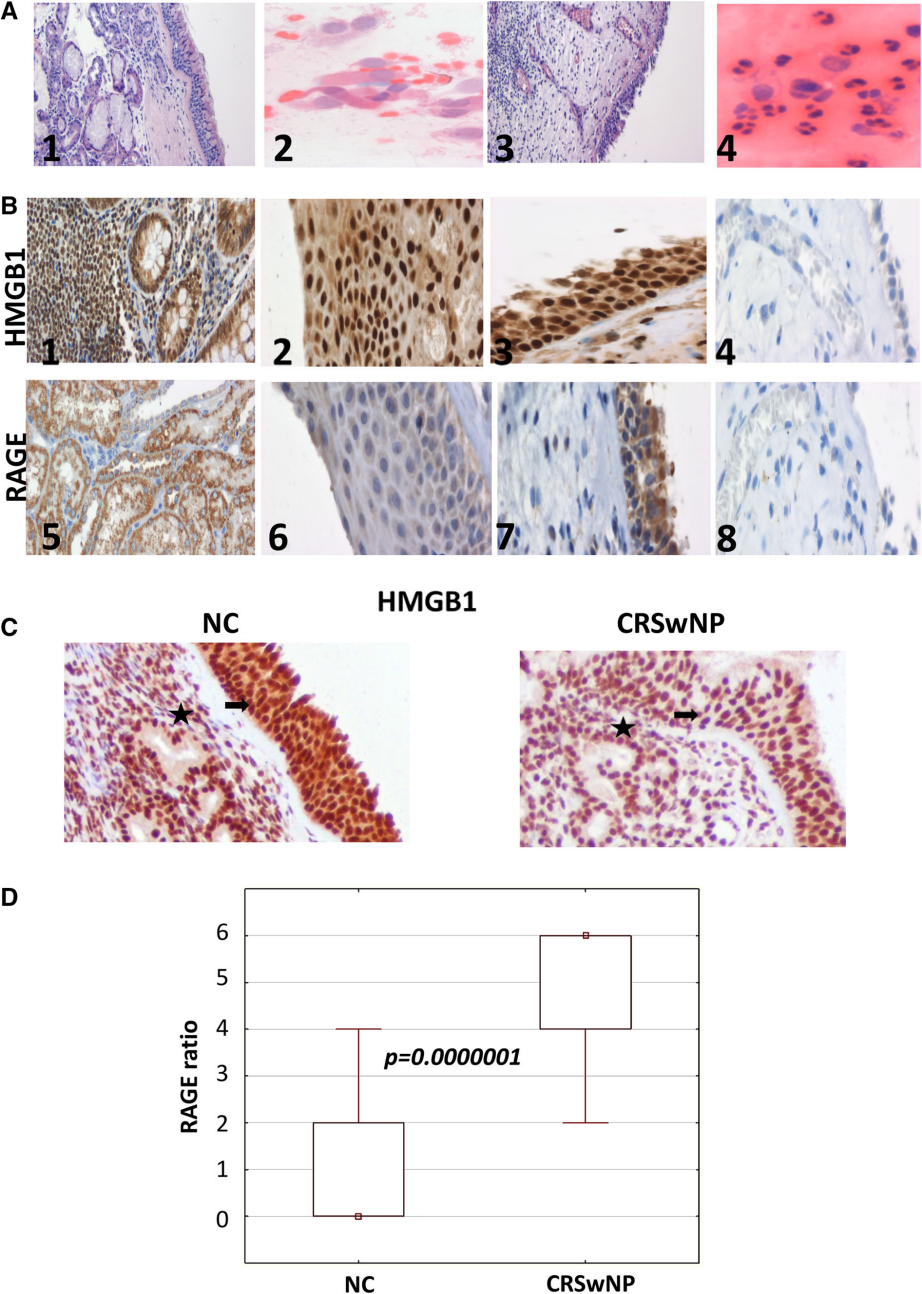
HMGB1 and RAGE expression in NC and in recalcitrant chronic rhinosinusitis with nasal polyps (CRSwNP) tissues. **a1** H+E staining of NC mucosa (×100); **a2** H+E staining of NC cytology taken under middle turbinate (×400); **a3** H+E staining of CRSwNP mucosa (×100); **a4** H+E staining of CRSwNP cytology taken under middle turbinate (×400); **b1** HMGB1 expression in the intestine (positive control; ×200); **b2** HMGB1 expression in NC tissue (×200); **b3** HMGB1 expression in CRSwNP tissue (×200); **B4** isotype control staining (×200); **b5** RAGE expression in the kidney (positive control; ×200); **b6** RAGE expression in NC tissue (×200); **b7** RAGE expression in CRSwNP tissue (×200); **b8** isotype negative control staining in CRSwNP tissue (×200). The sections were scored according to the percentage of rhinosinusitis tissue staining (positivity) (<25 % = 0; 25–75 % = 1; and >75 % = 2). The level of staining intensity was recorded as 0: none, 1: weak, 2: moderate, or 3: strong; as described in “Materials and Methods”). **c** Intensive HMGB1 immuno reactivity in both the epithelium lining nasal polyps (marked with *arrows*) and the stroma of nasal polyps (marked with *stars*). Representative pictures are shown. **d** Statistical analysis of RAGE expression in tissues. A *p* value of less than 0.05 was considered to be significant

In 25 cytograms taken from NPs, neutrophils predominance was found in 5 samples and eosinophils predominance in 20 samples. NC group showed a normal nasal cytology, characterized by the presence of many ciliated cells and muciparous cells, at the typical ratio 4–5:1. The results are consistent with the previously published data (Gelardi et al. 2009; Miszke and Sanokowska 1995). However, cytological data did not correlate with HMGB1 and RAGE expression (Fig. 1a).

### HMGB1 Expression in Tissue Sections

HMGB1 was detected in all NC and CRSwNPs tissues. HMGB1 was localized in the nuclei and cytoplasm and in all cases staining intensity was evaluated as moderate or strong (Fig. 1b). We did not find statistically significant differences between NC vs. CRSwNPs in terms of HMGB1 expression in epithelial cells of SM (*p* > 0.05; Fig. 1c). No correlation was found between HMGB1 expression and disease severity performed by nasal endoscopy examination, CT scan or number of prior surgeries. Furthermore, we did not find any significant differences in HMGB1 expression with respect to allergic or microbiological status.

### RAGE Expression in Tissue Sections

RAGE was detected in the cytoplasm of all patients with CRSwNPs and its staining intensity ranged from weak to strong. In contrast, RAGE was observed in the cytoplasm of only 40 % of NC patients, with the intensity of RAGE staining ranging from negative to weak (Fig. 1b). We found a significant difference in terms of RAGE expression in CRSwNPs vs. NC (*p* < 0.0000001; Fig. 1d).

### RAGE Expression Correlates with Disease Severity

**Fig. 2:**
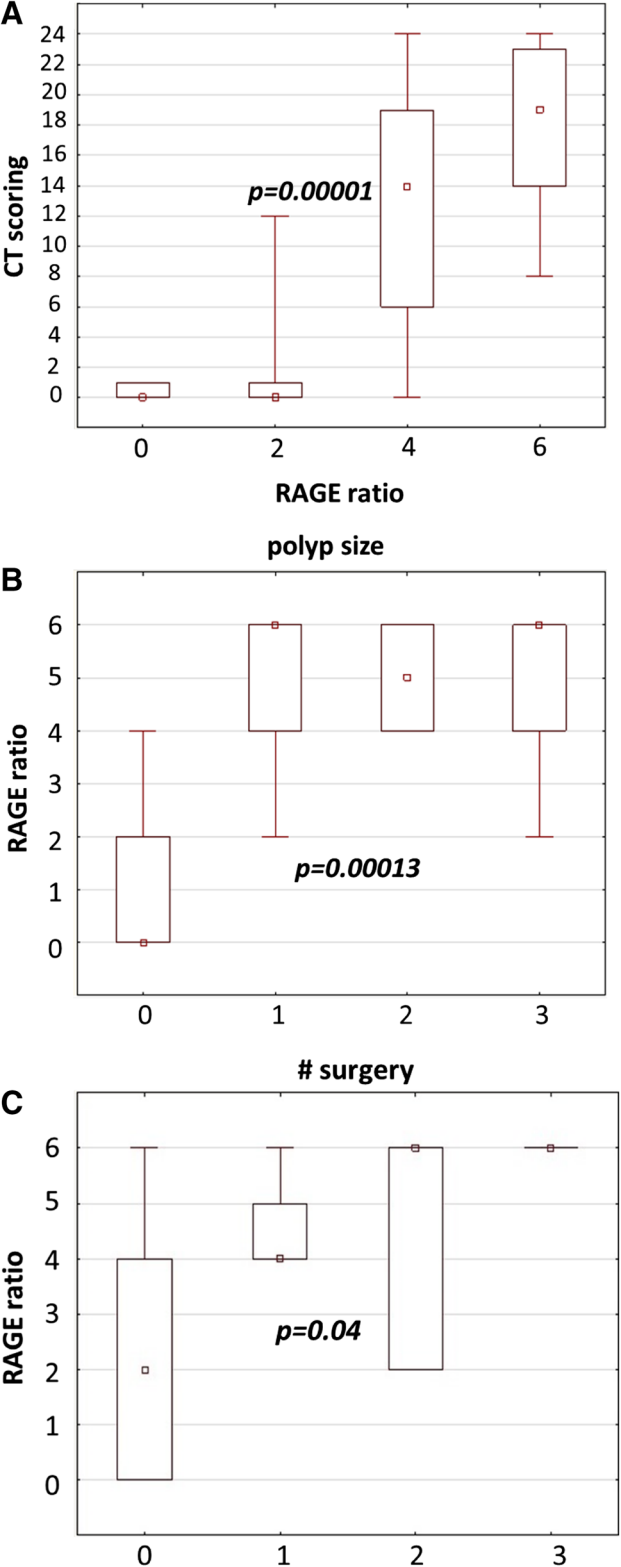
RAGE expression in correlation with disease severity. **a** RAGE ratio vs. CT scoring in both investigated groups; **b** RAGE ratio vs. polyp size; **c** RAGE ratio vs. number of previous surgery; *p* values were used to evaluate differences between CRSwNP patients and NC according to Bonferroni step-down procedure

In the CRSwNPs cohort, we observed significant correlations between RAGE ratio and severity of CT scan findings as graded by the Lund-Mackay scoring system. RAGE expression was stronger in patients with a higher CT score reflecting more extensive inflammatory changes in sinuses (*p* < 0.00001; Fig. 2a). We also observed significant correlation between RAGE ratio and the nasal polyp size noticed on endoscopy examination (*p* < 0.00013; Fig. 2b). RAGE staining also correlated with the number of prior sinus surgeries in the CRSwNPs group (*p* = 0.04; Fig. 2c).

### RAGE Expression Correlates with Disease Etiology

**Fig. 3:**
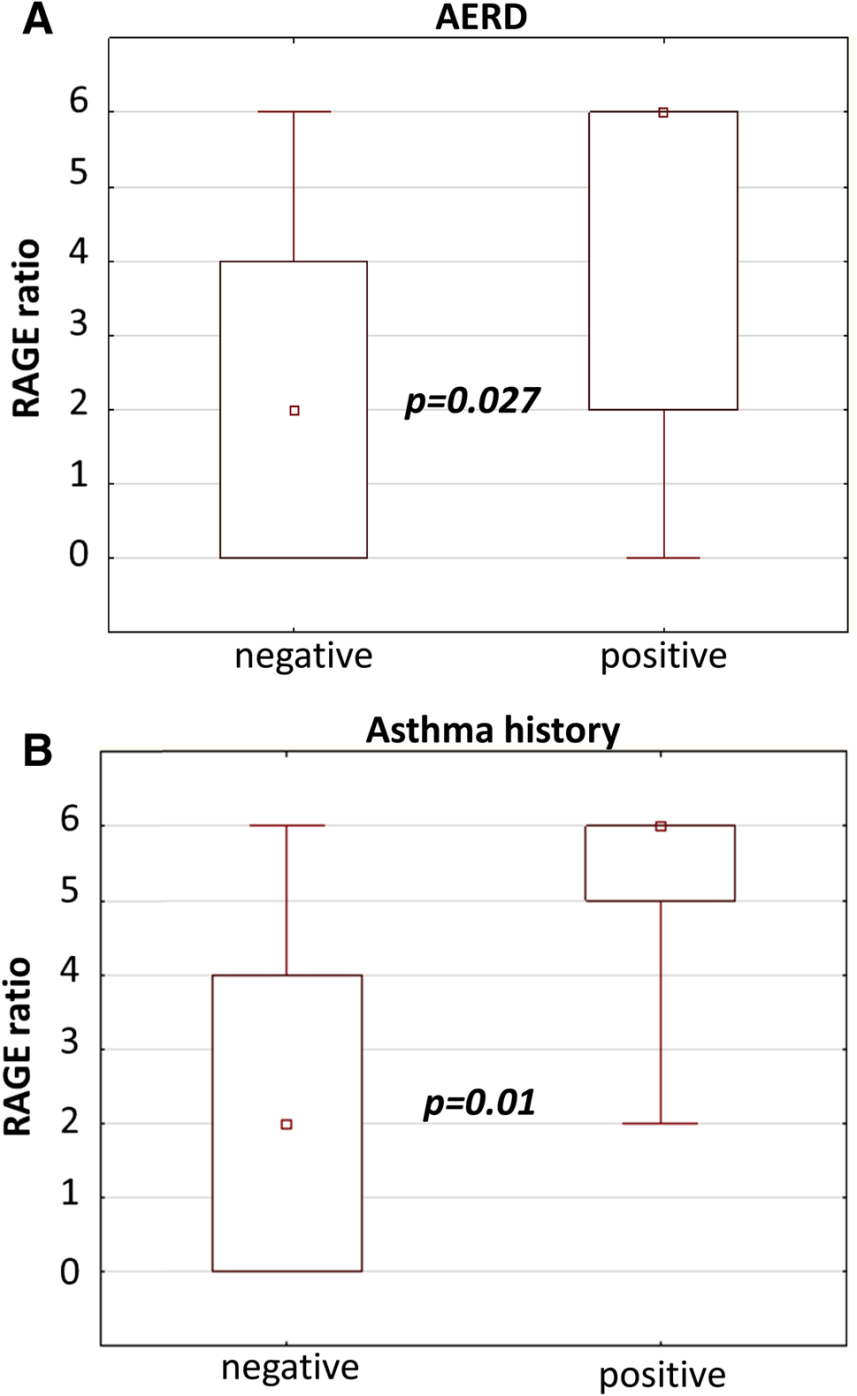
RAGE expression in correlation with asthma and AERD. **a** RAGE ratio vs. AERD; **b** RAGE ratio vs. asthma history

We observed significantly stronger RAGE expression in sinus mucosa of patients who suffered from asthma and AERD compared to other patients with negative anamnesis (Fig. 3). We did not observe any correlation between RAGE expression and allergy status or IgE titer. In the study, the microbiological status of nasal cavity and any specific microbe presence did not correlate with RAGE expression (*p* > 0.05).

## Discussion

A complex set of innate and adaptive immune pathways are active at the mucosal surface both constitutively and in response to specific antigens. Over activity or deregulation of the mucosal immune mechanism could lead to damaging persistent inflammation. To our knowledge, expression and function of RAGE in mucosal immunity of the sinonasal tract has not been explored. Currently, pathogenesis of CRSwNP is associated with aberrant innate or adaptive immune responses that may initially result from inappropriate expression of RAGE and HMGB1 protein or/and responses to its signaling pathway. To address this hypothesis we sought to understand the role of RAGE in recalcitrant CRSwNPs pathogenesis and the manner in which RAGE interacts with its ligand HMGB1 in the SM.

Here, we assess for the first time the expression level of RAGE in recalcitrant CRSwNPs and its target HMGB1 protein. In our study, HMGB1 was detected in all NC and CRSwNP tissues. HMGB1 was localized in the nuclei and cytoplasm, and in all cases staining intensity was evaluated as moderate or strong (Fig. 1b); no significant differences were observed between NC and CRSwNP patients (Fig. 1c). We investigated expression of HMGB1 in the whole tissue of NPs and mucosa, including the epithelial cells and the stroma of SM obtained from patients with CRSwNP vs. epithelial cells of nasal mucosa of NC. Available literature data indicated HMGB1 expression mainly in the inflammatory infiltrates (or polyps tissue) and not only in epithelial cells (Bellussi et al. 2012). In these studies, HMGB1 expression levels correlated significantly with eosinophilic infiltration and was higher in patients with large inflammatory cells infiltration in eosinophilic CRSwNP than in the controls. However, no significant differences in the HMGB1 protein level as well as the messenger RNA of HMGB1 were found between non-eosinophilic CRSwNP and controls (Chen et al. 2014). This observation is consistent with our findings.

RAGE was identified in the cytoplasm of CRSwNP patients more commonly than in NC patients, with consistently greater staining intensity in the positively staining subjects (Fig. 1b). Van Crombruggen et al. (2012) reported that RAGE is highly expressed in the human upper airways under normal physiology. In their CRSwNP patients, tissue levels of both soluble RAGE (sRAGE) and membrane-bound RAGE (mRAGE) were significantly lower versus the levels measured in control tissue. However, the relative levels in gene expression for mRAGE showed no difference between the CRSwNP patient and NC, while endogenous secretory RAGE was modestly but significantly higher in CRSwNP vs. control tissue. Nevertheless, some authors consider HMGB1 expression or RAGE separately and do not report HMGB1 and RAGE co-expression in their patients (Bellussi et al. 2013; Chen et al. 2013). For other authors, judging from the RAGE-DNA complex structure, it is clear that HMGB1 is not essential for the RAGE-DNA binding event, as DNA can bind to RAGE in the absence of HMGB1 (Sirois et al. 2013). This observation could be responsible for differences between RAGE and HMGB1 expression noticed in our study.

In our research, we found correlations between RAGE expression and disease severity including CT score, NP size and number of previous surgeries (Fig. 2). Based on these observations, we took under consideration the RAGE role in recalcitrant CRSwNPor recurrence after primary surgery. This correlation confirms a deregulation of the normal RAGE function as a consequence of the inflammatory and remodeling mechanisms involved in NP and furthermore suggests the implication of other factors in this process. Deregulation of RAGE and HMGB1 in the immune barrier function potentially compromises host defense and makes the sinus mucosa more susceptible to antigenic exposition, leading to chronic inflammation.

Thus in looking for potential etiology factors of CRSwNP, we assessed allergy, AERD, asthma and microbiologic status of nasal cavity for their correlation with RAGE and HMGB1. Shim et al. (2012) showed that HMGB1 expression was markedly higher in asthmatics than in NC and positively correlated with eosinophilic airway inflammation and non-specific airway hyper responsiveness. Furthermore, they found that enhanced RAGE expression on CD11b-CD11c(+) also significantly decreased when HMGB1 activity was blocked. In regard to this study, we observed significantly stronger RAGE intensity in sinus mucosa of patients who suffered from asthma and AERD compared to other patients with negative anamnesis but we did not find any correlation between RAGE expression vs. allergy status.

In addition to morphologic changes as a potential cause of the altered tissue levels of sRAGE, some authors reported increased colonization rate of *Staphylococcus aureus* in the upper airways of CRSwNP patients as a potentially novel contributor to the overall reduction in sRAGE in this patient group (Van Zele et al. 2004). Van Crombruggen et al. (2012) in ex vivo human tissue experiments showed that *S. aureus* was able to release free sRAGE into the tissue culture medium, otherwise sRAGE could be detected in the tissue-cube pellet only as tissue-associated sRAGE. In our study, however, the microbiological status of nasal cavity and any specific microbe did not correlate with RAGE expression (*p* > 0.05).

The potential pathogenic role of RAGE in other inflammatory disorders was supported by a few studies demonstrating that anti-RAGE/HMGB1 antibodies prevent chronic inflammation (Ostberg et al. 2010; Pisetsky et al. 2008). Application of this antibody could be effective in terms of improvement of the therapeutic repertoire for CRSwNP.

In conclusions, it is possible that the explanation for CRSwNP pathogenesis might be related to RAGE over expression with subsequent sinus mucosa hyper proliferation, recurrent NPs formation necessitating several operations. This observation potentially opens new ways for investigation of the RAGE function as one of the reasons for inflammation related to CRSwNP.

## Abbreviations

HMGB1: High mobility group box 1
RAGE: Receptor for advanced glycation end products
CRSwNP: Chronic rhinosinusitis with nasal polyps
NPs: Nasal polyps
AERD: Aspirin exacerbated respiratory disease
H+E: Hematoxylin-eosin
DAMPs: Damage-associated molecular patterns
EPOS: European position paper on rhinosinusitis
